# QT Assessment in Early Drug Development: The Long and the Short of It

**DOI:** 10.3390/ijms20061324

**Published:** 2019-03-15

**Authors:** Robert M. Lester, Sabina Paglialunga, Ian A. Johnson

**Affiliations:** 1 Cardiac Safety Services, Celerion, 2420 W Baseline Rd, Tempe, AZ 85283, USA; ian.johnson@celerion.com; 2 Scientific Affairs, Celerion, Tempe, AZ 85283, USA; sabina.paglialunga@celerion.com

**Keywords:** ICH E14, ICH S7B, cQT, exposure response, QT correction formulae, Fredericia, Bazett, short QT syndrome, long QT syndrome, QTc normal values, proarrhythmia, IKr blockade, CiPA, J-T interval, ventricular repolarization, Torsades de Pointes

## Abstract

The QT interval occupies a pivotal role in drug development as a surface biomarker of ventricular repolarization. The electrophysiologic substrate for QT prolongation coupled with reports of non-cardiac drugs producing lethal arrhythmias captured worldwide attention from government regulators eventuating in a series of guidance documents that require virtually all new chemical compounds to undergo rigorous preclinical and clinical testing to profile their QT liability. While prolongation or shortening of the QT interval may herald the appearance of serious cardiac arrhythmias, the positive predictive value of an abnormal QT measurement for these arrhythmias is modest, especially in the absence of confounding clinical features or a congenital predisposition that increases the risk of syncope and sudden death. Consequently, there has been a paradigm shift to assess a compound’s cardiac risk of arrhythmias centered on a mechanistic approach to arrhythmogenesis rather than focusing solely on the QT interval. This entails both robust preclinical and clinical assays along with the emergence of concentration QT modeling as a primary analysis tool to determine whether delayed ventricular repolarization is present. The purpose of this review is to provide a comprehensive understanding of the QT interval and highlight its central role in early drug development.

## 1. History of The QT Interval and Its Importance in Early Drug Development

In 1895, Einthoven developed the human electrocardiogram (ECG) and described the PR segment, QRS complex and the T wave while a decade later he and Lewis also recognized the existence of U waves [1]. In subsequent years, while T wave morphology changes were deemed pathologic and QT lengthening was noted in the setting of myocardial infarction, routine measurement of this interval was not deemed warranted. However, in 1957 Jervell and Lange-Nielsen identified a family in which 4 of 6 children had QT prolongation associated with deafness in the absence of any structural heart disease [2]. The affected persons were noted to have syncopal episodes and 3 of the children subsequently died before the age of 10 without any autopsy evidence of cardiac pathology. In the ensuing years multiple families were discovered with long QT intervals often without associated deafness, who had a proclivity to syncope and sudden death. In the early 1960s Romano and Ward [3,4] both described young individuals with long QT intervals and a history of fainting and ventricular fibrillation, frequently mediated by exercise and ameliorated by beta blockers. In light of these observations, cardiologists and other physicians became sensitized to the importance of evaluating the QT interval and the recognition that abnormal lengthening may predispose to serious ventricular arrhythmias and sudden death.

In 1964 [5], a small series of patients being treated with quinidine, was reported to have syncope due to ventricular tachycardia in the setting of a prolonged QT interval. The morphology of the ventricular tachycardia had a peculiar undulating or twisting appearance which in 1966 was termed Torsades de Pointes (TdP) by Desertennes [6]. In the subsequent decades, attention was consequently directed to routine measurement of the QT interval in patients receiving cardiac antiarrhythmic drugs such as procainamide, quinidine and disopyramide but routine surveillance for other classes of medicines was not performed as there was no known link between those agents and QT prolongation or TdP. The landscape changed in 1988, when prenylamine (Segontin) [7], a calcium channel blocking analogue of amphetamine used in the treatment of angina pectoris, was the first drug to be withdrawn from the market due to sudden death associated with QT prolongation. Several years later a case report of TdP and QT lengthening with the antihistamine terfenadine (Seldane) was published and this drug was eventually removed from the market in 1997 [8]. In the following years, additional classes of medications including antibiotics and psychotropic drugs were linked to TdP and a number of these agents were subsequently withdrawn by the Food and Drug Administration (FDA) [9]. In response to these events, government regulators and the pharmaceutical industry realized that a comprehensive evaluation of the QT interval and arrhythmia risk should be incorporated into a new compound’s development program.

The occurrence of life threatening ventricular arrhythmias in the presence of a prolonged QT interval also garnered the interest of British regulators who in 1999 drafted a paper entitled “Points to consider: the assessment of the potential for QT interval prolongation by non-cardiovascular medicinal products” [10]. This document was written in response to multiple reports of non-antiarrhythmic drugs prolonging the QT interval on the ECG resulting in TdP. It formed the basis for regulatory agencies worldwide to focus attention on the arrhythmic potential of drugs that were previously deemed to be safe.

Shortly thereafter Canada followed suit and developed a similar document in 2001 entitled “Assessment of the QT Prolongation Potential of Non Antiarrhythmic Drugs” [11] but no formal testing protocol was enumerated. In 2003 the International Conference on Harmonization (ICH) S7B document was published [12], which delineated the ***preclinical*** strategy to assess the potential of non-cardiovascular compounds to alter ventricular repolarization. This was followed in 2005 by regulators from the United States, Europe and Japan updating ICH S7B while also drafting the seminal clinical guidance document ICH E14 [13], which outlined recommendations to assess the arrhythmic risk of investigational human non-cardiac pharmaceuticals and their effect on ventricular repolarization. To date ICH S7B has not been formally amended, whereas ICH E14 has been updated with Q&A responses on multiple occasions in 2008, 2012, 2014 and 2015 [12] but has not been officially revised in entirety.

## 2. Overview of Ventricular Repolarization Electrophysiology

In order to properly understand the QT interval’s role in the assessment of ventricular repolarization as well as contextualize its evolution in regulatory guidance, a basic review of the cardiac action potential and principal ion channels is warranted.

**Figure 1:**
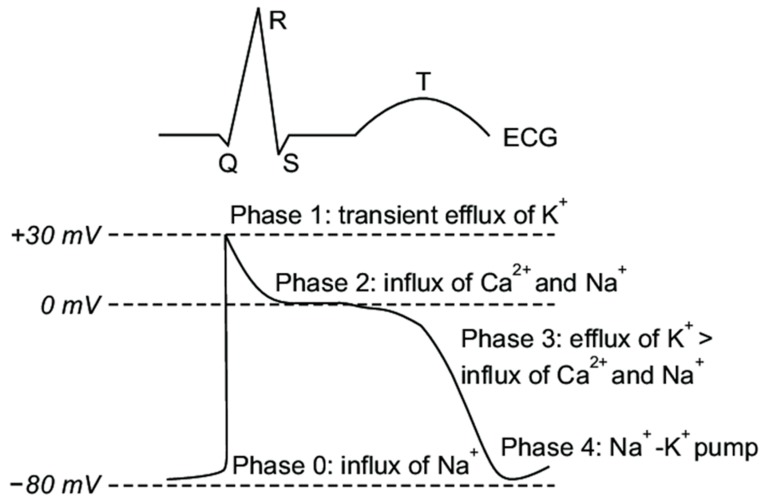
Cardiac Action Potential Phases. Major inward and outward cardiac ion channels affecting the five phases of the cardiac action potential. Note that these phases represent time dependent intervals based upon the ingress and egress of the various ions and their impact on transmembrane voltage. Reproduced from [14].

The electrical activity in mammalian cardiac cells is mediated by regenerative action potentials resulting from numerous voltage gated and ligand or receptor bound gated channels. These channels are proteins which change configuration so as to regulate the movement of various ions across the cell membrane. The channels may be open, closed or inactive and are dependent upon changes in myocyte membrane potential. Coordination of these action potentials in the ventricles is responsible for ventricular depolarization while their recovery and return to their baseline resting membrane potential results in repolarization. The cardiac action potential is divided into 5 phases as noted in Figure 1.

➢ **Phase 0:** The sharp upstroke of the action potential is primarily the result of a transient and rapid influx of Na^+^ (I_Na_) through opening of Na^+^ channels;
➢ **Phase 1:** The termination of the action potential upstroke and initiation of the early repolarization phase are mediated by inactivation of Na^+^ channels and the transient outward movement of K^+^ (I_to_) through K^+^ channels and chloride (Cl^−^) ions;
➢ **Phase 2:** The action potential plateau is ascribed to the balance between the influx of Ca^2+^ (I_Ca_) through long opening L-type Ca^2+^ channels and the efflux of repolarizing K^+^ currents;
➢ **Phase 3:** The sustained downward slope of the action potential and the late repolarization phase are due to the egress of K^+^ (I_Kr_ and I_Ks_) through delayed rectifier K^+^ channels;
➢ **Phase 4:** The resting membrane potential is supported by the inward rectifier K^+^ current (I_K1_), the sodium potassium ATPase pump, and the Na^+^/Ca^2+^ exchanger.

Potassium channels are the most abundant of all mammalian ion channels. There are four physiologic subtypes: those which are calcium mediated, those which are known as tandem pore domain channels seen in neurons, those which are inwardly rectifying, and those which are voltage gated. The latter two subtypes are the main channels governing cardiac cell function (reviewed in [15]). Voltage gated cardiac potassium channels are pores in the membrane of excitable cells that open and close due to alterations in transmembrane voltage and electrochemical gradients thereby allowing the egress of potassium out of the cell. Of the multiple genes that have been identified that encode potassium currents, the inward rapidly delayed potassium rectifier current I_Kr_ encoded by the human ether a-go-go related gene (hERG) on chromosome 7 termed KCNH2 and the slowly activating delayed rectifier current I_Ks_ encoded by KCNQ1 and KCNE1 genes are the biggest contributors that influence action potential duration (APD) [16,17]. The inner region of the potassium pores is dense with aromatic and polar residues, which act as promiscuous target binding sites for both cardiac and non-cardiac pharmacological drugs [18]. Genes that encode the potassium channels are responsible for the formation of both alpha and beta subunits which combine into hetero-oligomeric complexes that affect the gating function of these channels. Blockade of the alpha subunit which carries the I_Kr_ current is the most common mechanism by which pharmaceuticals prolong the action potential resulting in QT lengthening which forms the basis for TdP.

In the absence of dispersion of depolarization, changes in phase 3 of the action potential are primarily responsible for QT prolongation and increases in the effective refractory period of cardiac tissue. This lengthening, in turn, may be related to modification or blockade of various cation voltage gated channels. To illustrate, prolongation of the action potential can result from inhibition of one or more of the outward K^+^ currents, decreased inactivation of the inward Na^+^ or Ca^2+^ currents, increased activation of the Ca^2+^ current, alteration of potassium channel trafficking or disruptions in protein synthesis [19], see Figure 1. Less commonly, QT lengthening may also be mediated by drugs such as quinidine that prolong APD and the effective refractory period of cardiac cells via blockade of the fast inward Na^+^ current channels corresponding to the upstroke (phase 0) of the action potential. The resultant impact on cardiac tissue is to produce a transmural gradient of repolarization which provides the substrate for re-entrant arrhythmias.

## 3. How Is the QTc Calculated: Popular Correction Formulae for QT Values

There is typically an inverse relationship between the heart rate (RR interval) and the QT interval with shorter QT intervals at higher heart rates and longer QT intervals at lower heart rates. As such, multiple efforts have been advanced in an attempt to correct for this relationship throughout a range of heart rates ideally demonstrating that the regression line comparing the corrected QT (QTc) value against the RR interval is a flat line with a slope of zero. In 1920 Bazett determined that the duration of ventricular systole on the ECG was related to the square root of the heart rate and proposed a correction formula based upon measurements in only 12 subjects [20], see Table 1. In the same year, Fredericia posited that this relationship was a function of the cube root of the RR interval in a small sample of ECGs from a presumably normal population of individuals [21]. Since then numerous correction formulae involving exponential, linear, and logarithmic equations have been advanced in an effort to identify which formula optimally negates the effect of heart rate on the QT interval. In general, each of these population-based formulae using various mathematical models were applied to the dataset rather than allowing the dataset to drive the best fit relationship between the heart rate and QT interval. Moreover, these mathematical models were derived from heterogeneous populations of individuals thereby contributing to a lack of congruity especially when there is heart rate variability. As such, the most common population-based formulae have all demonstrated that the plot of QTc vs. the RR interval is usually imperfect with positive slopes that do not completely eliminate the impact of heart rate. As such, a single mathematical formula may not be appropriate to account for the QT-RR relationship when there is significant heart rate variability.

Historically, the most commonly used correction formula is Bazett which over the years was integrated into the output of most standalone digital ECG acquisition carts and is still utilized today by many clinicians worldwide. It is most accurate between heart rates of 60–100 beats per minute (bpm). At slower heart rates less than 60 bpm, the formula undercorrects the QTc value while at values over 100 bpm the formula overcorrects the QTc interval. The 2005 ICH E14 guidance document originally relied upon the Bazett calculation for reporting of cardiodynamic and safety ECG data in early clinical trials. Subsequently, due to the shortcomings noted above, Bazett’s corrected data has been shown to be inferior to the Fredericia correction method and is no longer routinely warranted for reporting by the FDA (Q&A from FDA reference) [29]. It still, however, does enjoy a primary role in screening and management of patients with congenital long QT syndromes especially in the pediatric population. To amplify this point, there is some evidence that in a very young pediatric population, Bazett’s formula best corrects for heart rate. In support of this proposal, Phan et al. [30] interrogated a database of 702 children under 2 years of age being screened for long QTc syndrome and compared regression slopes of heart rate to QTc employing Bazett, Fredericia, Hodges and Framingham formulae (Table 1). In this specialized cohort, Bazett proved to yield the most consistent calculation of QTc and the upper limit of normal value (ULN) was 460 ms which coincides with that recommended for congenital long QTc screening in this population.

Fredericia’s correction (QTcF) is currently the standard adopted by the FDA when submitting QT data for review. When there is suboptimal QTc correction by Fredericia, consideration should be given to use an individual correction factor especially when there is some variability in heart rate as described below. This approach requires determining an individual exponent that best eliminates the impact of heart rate on the QT interval. The magnitude of variability that has been proposed by regulators to perform this calculation is a documented heart rate change of 6–10 beats per minute. Morganroth [31] recommended a number of ECGs under non-treatment conditions for each individual be obtained across a sufficient range of heart rates (35 to 50 ECGs covering a range of heart rates from 50 to 80 bpm) in order to adequately make this calculation. Zareba and coworkers [32] have published data recommending that at least 400 predose QT-RR pairs for each individual subject are needed to compute an acceptable individual correction. Malik recently presented his viewpoint that QT/RR regression analysis in small cohorts of subjects as seen in early phase human studies is fraught with both false positive and false negative conclusions due to significant inter-individual variability [33,34]. To add, he stated that a single mathematical QT/RR relationship that fits for all persons individually is unobtainable and therefore should be derived separately for each subject in a clinical trial. He further commented that the individual correction factor is universally superior to population-based formulae although this position is not universally supported.

When electing to perform an individual correction (QTcI), one popular approach for this calculation is to obtain a full day of Holter recording at baseline prior to any treatment (Day −1) and then examine all evaluable QT-RR pairs to calculate this correction factor according to the following protocol: All QT/RR pairs from each subject are used for that person’s individual correction coefficient, which is derived from a linear regression model: log(QT) = log(a) + b × log(RR). The coefficient of log(RR) for each subject, b_i_, is then used to calculate QTcI for that subject as follows: QTcI = QT/RR^bi^. Also, the effects of any potential hysteresis needs to be an important adjunct to this calculation [35].

There are a number of additional methodologies as to how to calculate QTcI in the presence of an increased heart rate as reported by Garnett et al. [35]. These authors reviewed the relative strengths and weaknesses of alternative techniques including Holter bin analysis, beat to beat comparisons and one stage fixed effect pharmacodynamic-pharmacokinetic modeling although there was no consensus as to which of these approaches was optimal. They further discussed the role of exercise and autonomic maneuvers including postural changes designed to augment the heart rate although these latter protocols are not standardized and routine acceptance has proven elusive.

Most recently, using a large US National Health and Nutrition Survey (NHANES) database of subjects from the general population, a cross validated spline correction factor was reported which essentially “obliterated” the relationship between the QTc and RR intervals and this effect was independent of gender [27]. The authors also compared this correction factor to 6 other well-known methods and found that the spline method showed superior performance. They deemed their approach “heart rate agnostic” as the regression lines of heart rate vs. QTc interval were virtually flat in all cases. Adoption of this novel approach, while intriguing, must await further validation and reproducibility in additional studies involving large populations of healthy subjects encompassing a spectrum of heart rates and ages.

## 4. What Is a Normal QTc Value

There is considerable debate as to what constitutes a prolonged QTc interval and there have been a large number of published studies on this subject over many decades. In general, the studies are not homogeneous as some included manual caliper measurement on paper while others involved the more precise method of utilizing on screen digital calipers with digitally acquired ECGs. To add, some protocols entailed measuring a single complex in a single lead, multiple complexes in a single lead and then averaging the results, choosing the longest lead on the 12-lead tracing, or using a representative global median beat for QTc determination. Moreover, studies were not always performed in healthy volunteers and were comprised of populations with different age cutoffs as well as those who may have had underlying cardiovascular or other clinical pathology. Lastly, not all studies employed the same QT correction method when reporting results although correction formulae for the lower limits of normal (LLN) values were generally consistent and independent of gender while those for the ULN values showed substantial variation [20].

In 2006, Goldenberg and colleagues examined 581 subjects of varying ages and found the ULN threshold for men was 450 ms while for women it was 470 ms [32]. Thereafter, in 2009 Rautaharju et al. published the American Heart Association (AHA)/American College of Cardiology (ACC)/Heart Rhythm Society (HRS) recommendations for normal QTc values which have been the most widely accepted with healthy males having an ULN value of 450 ms while for females the value was 460 ms [36]. In contrast, according to the AHA expert panel report by Drew and colleagues [37] on prevention of TdP, a prolonged QTc should be defined as a value that exceeds the 99th percentile of the population in question. For healthy postpubertal males this was 470 ms and for females this was 480 ms. Reijinbeek et al. studied 13,354 individuals ranging in age from 16–90 years and evaluated 12 lead QTc intervals employing a broad array of different correction formulae with a value exceeding the 98th percentile considered abnormal [38]. They found that the ULN for men was 460 ms while for women it was 470 ms employing Bazett’s correction which was routinely 10–15 ms higher when compared to the Fredericia formula. They also determined that the QTc value increased slightly with age and was always higher in women by around 10 ms independent of the correction factor chosen. One criticism of this study is that it was a meta-analysis of data from 4 other trials which included heterogeneous populations of both healthy individuals and those with underlying disease.

Mason et al. derived reference ranges from 79,743 subjects enrolled in clinical drug trials with ages ranging from 3 months to 99 years [39]. Some of these individuals did have cardiovascular disease or other pathology and the QTc was determined using both single lead median beat measurement as well as taking the average of 3 complexes measured in the lead with the longest QTc value. There were a total of 21,457 males and 24,562 females with suitable ECGs for QTc analysis using digital on screen calipers. They employed the 98th percentile for the ULN threshold and found that for men that value using Fredericia’s correction was 438 ms while for women the number was 450 ms. The corresponding QTcF LLN values were 355 ms for men and 365 ms for women. A final report of interest is that of Olbertz and colleagues who reviewed a cohort of 39,303 ECGs from healthy volunteers using ULN cutoffs at the 97.5th percentile while the LLN cutoff was at the 2.5th percentile [40]. They showed that the ULN for the entire population was 450 ms with females exhibiting an approximately 11 ms longer QTcF than males. The LLN cutoff for the entire population was <350 ms and there was no significant gender difference for this determination.

The decision as to which set of criteria should be utilized depends upon the goal of screening. To best identify individuals from the general adult population who may have congenital long or short QTc syndromes, LLN values of <330 ms and ULN values of >460 ms have been suggested by Ackerman [41]. In contrast, for early clinical trials there is a tradeoff of using lower ULN values to reduce the risk of the QTc reaching a threshold of regulatory concern during study conduct while offering a better margin of subject safety, versus a higher QTc value which would facilitate the recruitment of healthy volunteers. In this regard, a value of >450 ms irrespective of gender as originally suggested by the FDA in their ICH E14 guidance document is most often utilized in first in human protocols [12]. Alternatively, given the known gender differences, a cutoff value of >450 ms for males and >460 ms for females as per the AHA/ACC/HRS consensus document would also seem reasonable for screening in early phase clinical trials. Whatever numbers are chosen, these thresholds should be reviewed with sponsors when designing protocols and modified when appropriate depending upon preclinical data, the compound under investigation, and the study design, acknowledging that subject safety is always paramount in these discussions.

## 5. Measurement of the QT Interval 

**Figure 2:**
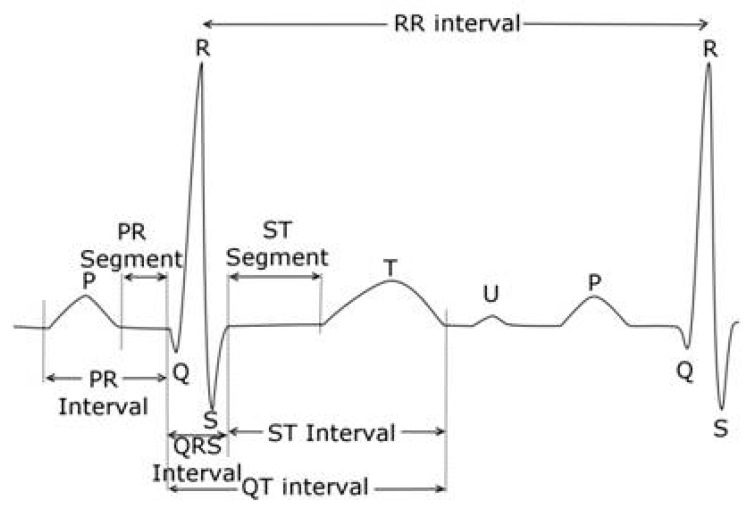
Electrogradiogram (ECG) Principal Waveforms and Intervals. Reproduced from [44].

The QT interval is measured from the beginning of the QRS complex which depicts the surface representation of the initiation of ventricular depolarization acknowledging that some myocytes will begin repolarizing during this phase of the cardiac cycle. The end of the T wave corresponds to the surface representation of the termination of ventricular repolarization (reviewed in [42,43]), and shown in Figure 2. Ventricular repolarization in turn, is primarily demarcated from the end of the QRS or J point to the end of the T wave, also referred to as the J-T interval. Measurement can be performed on multiple QT complexes in a single lead, on a single lead median beat, on a 12-lead representative complex or on a computer generated 12 lead global composite median beat.

The ECG complex(es) used for interval measurement is not uniform and differs between core laboratories. When using a single lead for analysis, controversy exists as to which lead or leads to utilize for measurement since the QT interval can vary by over 20 ms within a single lead or between leads in a 10 s tracing even in the presence of stable sinus rhythm [45]. Furthermore, an individual’s QT and QTc demonstrates diurnal variability with the longest values occurring during sleep and the shortest values occurring during the day. This individual variability due to circadian rhythm was reported to be as high as 95 ± 20 ms by Molnar and associates in a small group of 21 subjects with the longest QT being recorded just after awakening [46]. In addition, the QT interval adapts to changes in heart rate with a delay known as QT hysteresis which may not be evident for several minutes, thereby underscoring the importance of measurements during periods of heart rate stability and in the absence of artifact or arrhythmias. As such, it is imperative in assessing the impact of drugs on the QT segment in the same lead or using the same composite lead methodology and at the same time of day during the study (time matched). Finally, standard and rigorous acquisition procedures should be adopted to control for the role of food, sleep, body position, gender, autonomic tone, electrolyte abnormalities and environmental factors all of which can influence the QT measurement.

For decades, the QT interval was measured on a 10 s gridline paper ECG by manual caliper placement at a paper speed of 25 mm/s in which a 1 mV calibration pulse signal produced a 10 mm deflection. Higher paper speeds of either 50 mm/s or 100 mm/s were occasionally used to provide more accuracy especially when there was a need to discern the presence of heart block or arrhythmias. However, these higher recording speeds are not routinely advised as they can lead to distortion of U waves and other low amplitude waveforms thereby confounding accurate interval measurement.

Prior to the 2005 ICH E14 guidance document, 3 QT complexes were usually assessed in lead II and the three measured intervals were then averaged to provide a mean QT value. Thereafter, the QTc was calculated from this number based upon the associated RR intervals bracketing each complex. The rationale for selecting lead II for measurement was rooted in a number of factors. In the past, Bazett used lead II for QT analysis since at that time ECGs were devoid of any precordial leads and this therefore became the *defacto* choice for many years. Secondly, in the majority of children with congenital long QT syndromes, lead II was recommended as it usually yielded the longest QT result in this group. Lastly, lead II has been the most popular lead since the P, QRS and T wave vector axes are mainly directed inferolaterally towards that lead and confounding U waves are not usually evident or as prominent as in the precordial leads.

**Figure 3:**
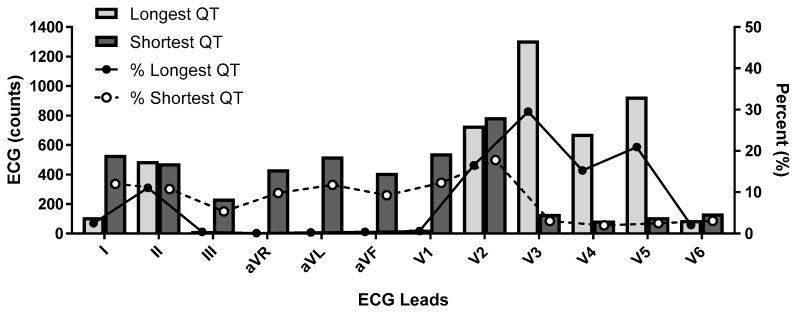
Shortest and Longest QT interval by Lead Selection.ECG lead data obtained from 4429 participants. Distribution of shortest and longest QT interval by lead in counts (*x*-axis) and as a percentage (*y*-axis).

The 2009 AHA/ACC/HRS article states that the QT should be measured in the lead demonstrating the longest interval and they suggest using leads V2 and V3 as these leads tend to have earlier QRS initiation compared to the limb leads [36]. This recommendation is somewhat contrary to findings from the observational study of 4429 volunteer subjects enrolled in clinical trials (RML personal observation), where the shortest QT intervals were noted in leads I, V1, and V2 while the longest intervals were seen in leads V3, V4 and V5 (Figure 3).

However, other authors have argued that midprecordial leads should not be used as they may overestimate the true QT interval and they suggest using either leads II or V5 which remain the most commonly employed by ECG core laboratories as well as in the congenital long QT registry. Lead II and a “precordial lead” are also those that were initially recommended by the FDA in their 2005 guidance and are therefore widely accepted for regulatory submission.

FDA guidelines from the 2005 ICH E14 and Q&A R3 documents states that ECGs should be measured by a “few skilled readers” ideally in a centralized core laboratory. They advocated for measurement by either a fully manual or a manual adjudication methodology in which computers initially placed the interval markers which were then adjusted by the skilled reader. In view of the variability between different algorithms and equipment manufacturers, fully automated measurements were only deemed appropriate for later phase trials with compounds that did not reveal any QT liability concerns in preclinical or first in human studies.

An important area for discussion in this recommendation is that the FDA left open who should be considered a “skilled reader.” A landmark paper by Viskin et al. found that the majority of physicians could not identify a long QT interval when one was present [47]. They provided 2 normal ECGs and 2 ECGs from patients with long QT syndrome to a worldwide group of physicians including 25 QT experts, 102 arrhythmia specialists, 332 cardiologists and 442 non-cardiologists and asked them to classify the QT as normal or abnormal. Ninety-six percent of QT experts correctly identified the QT intervals as long or normal, whereas only 62% of arrhythmia experts and less than 42% of cardiologists and non-cardiologists were correct in their QT diagnoses. This study underscores the importance of utilizing a centralized core laboratory with highly experienced pharma QT experts as the “skilled reader” pool. Furthermore, these readers should ideally be blinded to the treatment, time and all subject identifiers when interpreting ECGs in clinical trials.

Another factor of considerable significance is that intra- and inter-reader variability should be minimized and preferably established for each clinical trial while core laboratory quality metrics should be periodically obtained and tabulated to ensure reader certification and maintenance of their reading skills. As a standard operation procedure, when reading studies, assigning a single reader per subject or, if feasible, a single reader to the entire trial would optimize consistency and reduce variability. In this same vein, a comprehensive lexicon of diagnostic statements with associated definitions and criteria should be adopted by the core laboratory to establish uniformity in diagnostic interpretation and measurement. In core laboratories where the skilled readers only review computer generated outliers, it is further suggested that a 2% or greater blinded sampling rate of computer generated “inliers” should be procured and sent to the skilled reader(s) on a study specific basis to ensure the output of quality data.

As previously mentioned, the initial ICH E14 document stated that all ECGs in a clinical trial could be measured by the skilled reader pool using “manual adjudication” with or without computer assistance. In view of the high cost and considerable time involved in this scheme and pursuant to the 2014 FDA Questions and Answer (Q&A R2) guidance, there has been a shift away from using “skilled readers” to manually review all ECGs towards the use of computer assisted ECG algorithms [12]. These algorithms are designed to accelerate drug development timelines at reduced expense while maintaining data quality and integrity. These computerized platforms have been validated, their measurements are not statistically different from manually adjudicated ECGs, and datasets from these highly automated systems have been successfully submitted and accepted by the FDA for New Drug Applications (NDA). With the advent of on-screen digital calipers in combination with digital ECG acquisition, the ability to measure the QT interval precisely and accurately has dramatically improved. Today there are a number of software programs that permit the reader to evaluate this interval in a variety of presentations most commonly in a single lead II median beat format or in a representative superimposed 12 lead median beat. Finally, the approach of analyzing a limited number of single complexes may soon be supplanted by technology which permits automated measurement of all representative complexes over a 5 min or longer period of time so as to provide a more accurate temporal representation of the QT interval and repolarization lability.

**Figure 4:**
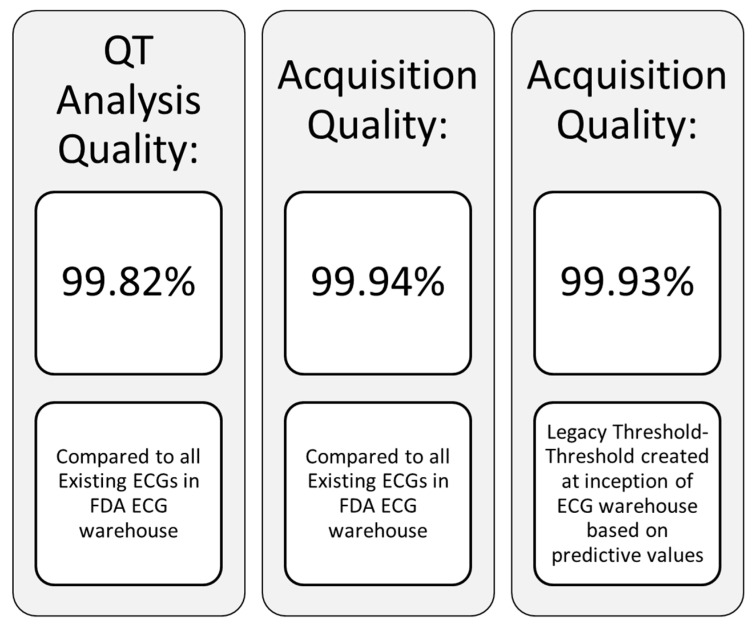
ECG quality metrics.

An essential point to clarify is the fundamental difference between safety and cardiodynamic ECGs produced in clinical trials both of which may be obtained from the same digital recording device. The former are typically single replicate 12 lead 10 s ECG tracings secured for screening of subjects prior to enrollment, again prior to drug dosing, and periodically after dosing so as to enable ***real time review*** by the principal investigator to evaluate subject safety. Safety ECGs are also used in conjunction with clinical and laboratory information as an integral part of dose escalation decisions. In contrast, cardiodynamic ECGs are also 12 lead 10 s tracings that are typically obtained over a 24 h period from a continuous Holter recording device at specific timepoints coinciding with pharmacokinetic drug determinations. These ECGs are most commonly extracted in triplicate (or higher multiples) over a 5 min time window when subjects have been supine for at least 10 min with careful attention directed at minimizing any environmental stimuli that might affect data quality. The extracted cardiodynamic ECGs are then processed and blindly ***reviewed at a later time*** by the ECG core laboratory readers to determine if the test compounds has any impact on ECG morphology or fiducial intervals related to cardiac conduction and ventricular repolarization. This information is then sent to the sponsor in a Statistical Analysis System (SAS) validated dataset for their review. The waveform data is concomitantly sent to the FDA warehouse in an HL7-XML format where the FDA provides ECG quality metrics to the core laboratory [13], a representative excerpt of which is noted in Figure 4. An added benefit of the government’s ECG warehouse is that it serves as an open access repository for research projects by all stakeholders to further the science in this field.

The traditional approach advocated by the FDA for cardiodynamic ECG sampling was to obtain ECGs in triplicate and this recommendation has been supported by several analyses both of which showed that obtaining more than 3 ECGs at each nominal timepoint yielded decreasingly small benefits in QT variability at increased cost [48,49]. However, within the past several years, there has been growing attention centered on the methodology known as high precision QT analysis [50], which involves extracting up to ten 10 s 12 lead ECGs at each protocol specific nominal timepoint as a means to enhance measurement precision. While this approach may indeed improve precision compared to conventional triplicate ECG tracings, it is unclear if the additional data points significantly reduce reader variability, justify the higher costs of this strategy, enhance the ability to detect a QT signal, or improve the likelihood that a new compound will be approved. Moreover, the FDA does not endorse any specific methodology, certification process, or core laboratory for early clinical trials nor have they mandated that more than triplicate ECGs be obtained to assess proarrhythmic risk. Thus, the advantage(s) of obtaining higher numbers of replicates remains controversial.

## 6. Problematic and Challenging Issues in QT Assessment

Measurement of the QT interval can prove quite challenging and may be affected by multiple ECG abnormalities. As a general rule, if the QT interval is less than half of the RR interval in sinus rhythm, then the QTc is probably within normal limits and less than 460 ms. Another caveat is that computer measurements should not be accepted as being accurate and careful inspection and manual adjudication are recommended whenever the computer-generated values are abnormal. This is especially true in the setting of any of the ECG findings enumerated below. To add, it is imperative that all manually adjudicated QT measurements be executed in a highly magnified view with 1 ms resolution to enhance accuracy. This is especially critical given the FDA’s 10 ms QTc threshold of regulatory concern and their requirement to submit individual categorical QTc analyses for values that deviate by both 30 ms and 60 ms from the baseline determination or breach a value that would be classified as an adverse event.

➢ **Artifact:** Measurement of the QT interval should be performed in tracings without any artifact that may obscure the intervals and lead to erroneous values. As such, a segment of the extracted ECG devoid of artifact should be used for measurement or additional “clean” ECGs as close to the nominal timepoint specified in the protocol time and events schedule should be secured and used for interval assessment. To aid in this regard there are automated computer algorithms designed to ensure that extracted cardiodynamic ECGs are obtained without significant artifact, dysrhythmias or heart rate instability.
➢ **U waves**: U waves are common especially in young individuals with relatively slow heart rates and often are distinct positive waves after the T wave and best seen in leads V2 and V3 (Figure 2). They are thought to represent a final phase of ventricular repolarization involving the summation of early afterdepolarizations or repolarization of mid myocardial M cells, papillary muscles or purkinje fibers [51]. U waves may be attenuated with filtering or indistinct when there is significant tachycardia. The U wave should be clearly identified as distinct from the T wave and should not be included in QT measurement as normal QU values have not been established and inclusion of the U wave would lead to gross over-measurement of the QT interval. When it is unclear if U waves are present, inspection of neighboring leads on the ECG may be helpful in separating a discrete U wave from the T wave.
➢ **Bifid T waves**: T waves may be notched or bifid in appearance and the end of the T in these cases should be measured after the second peak. Also, careful inspection of notched T wave morphology and the distance between notches may be useful in distinguishing the T wave from a superimposed U wave. While differentiating a notched T wave from a superimposed U wave can be difficult, viewing alternate leads in the tracing should be performed to help make this distinction.
➢ **Flat T waves**: When the T waves are flat, measurement of the QT interval should be carried out in a lead(s) where the T waves are positive, monophasic and best defined. In the absence of any positive unidirectional T waves, a clearly visible monophasic negative T wave would also suffice for this assessment. When a single lead median beat approach is being used and the designated lead is not suitable for measurement, the alternate lead utilized should be identified on the report and subsequent ECGs from that subject should all be measured in that same lead in order to provide procedural consistency and reduce variability that may be misconstrued as drug mediated.
➢ **T-U fusion**: U waves may be fused with the T wave thereby artificially prolonging the QT interval by casual visual inspection. In this setting, as initially recommended by Lepeschkin and Surawicz [52], a tangent line should be drawn through the steepest portion of the T wave downslope until it intersects the isoelectric line (defined by the T-P segment) and that crossing point is to be designated as the end of the T wave.
➢ **Arrhythmias**: Whenever the RR interval shows significant variability as in the case of sinus arrhythmia or atrial fibrillation, multiple evaluable QT complexes should be measured and the QT value averaged for all complexes so as to avoid over or under estimation of the QT interval. In the case of premature supraventricular or ventricular beats, measurement in the complex immediately following the premature beat should be avoided as ventricular repolarization is altered in the complex after a premature beat.
➢ **Asymptotic prolonged downsloping T waves**: This is a finding in which the QT interval can easily be overmeasured due to a prolonged T wave “tail”. As such, when the end of the T wave approaches the isoelectric line asymptotically, a tangent function utilizing the steepest portion of the downslope of the T wave should be employed as described for T-U fusion.
➢ **Wide QRS complexes**: The presence of a widened QRS such as with bundle branch block, ventricular pacing, pre-excitation, or intraventricular conduction delays, may contribute to a prolonged QTc interval which may not be a consequence of significantly altered ventricular repolarization. In these circumstances, the formula QTc = measured QTc-(QRS-100 ms) has been suggested to provide a clinically useful determination of the true QTc interval [41]. This approach has been advocated by those involved in assessing individuals with suspected or known congenital long QTc syndromes.
➢ **Misconnected limb leads:** In cases where there is limb lead misconnection involving only reversed arm leads, measurement can still be affected in lead II if a single lead median beat approach is used. In cases where lead II is effected by misconnected limb leads, an alternative precordial lead such as V5 is suggested. Misconnected limb leads should not significantly alter the QT measurement when a representative 12 lead median beat is utilized.

**Figure 5:**
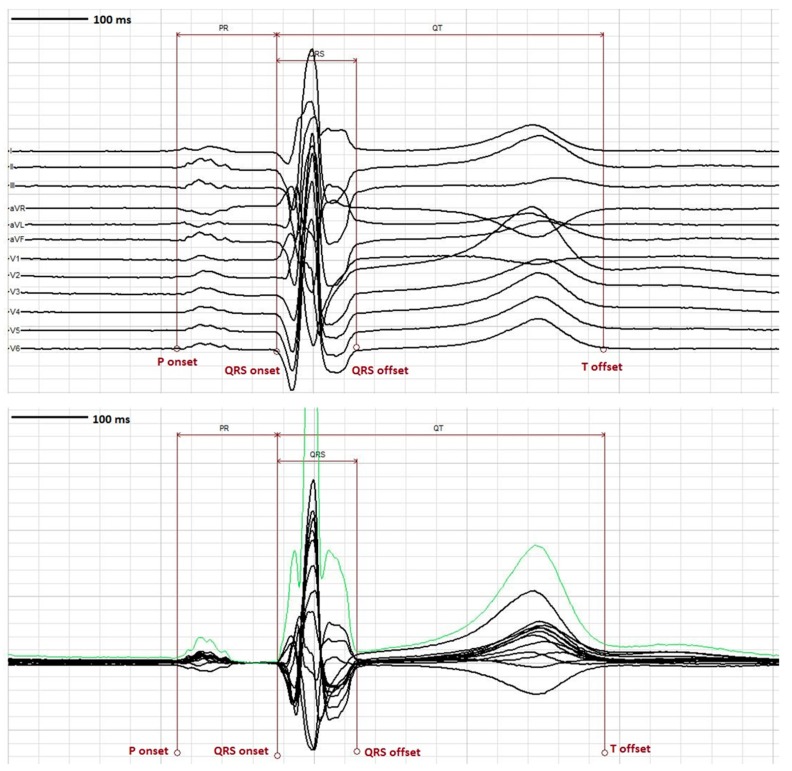
ECG tools. Top panel- Representation of 12 lead median beat using the “vertical separation” tool to enable better visualization and accurate placement of fiducial markers at the onset and offset of PR, QRS and QT intervals. Bottom panel- Representative 12 lead median beat with “vector magnitude” overlay in green which can facilitate accurate placement of fiducial markers at the onset and offset of PR, QRS and QT intervals.

Some computer software such as Cal ECG from Analyzing Medical Parameters for Solutions (AMPS) provides additional measurement tools to aid in determining the onset and offset of the PR, QRS, QT and J-T intervals. These include the ability to vertically separate all 12 leads when a representative median beat is used for measurement as well as the use of a superimposed vector magnitude display (Figure 5). These tools in the hands of a QT expert may assist in improving precision and accuracy of all fiducial markings compared to systems that lack these capabilities. Another alternative involves assessing the QT/RR relationship over longer time periods to better account for possible hysteresis due to dynamic and autonomically mediated heart rate effects. Finally, there have also been advocates for using either a dynamic beat to beat or a Holter “bin” method to determine the QT interval which has its greatest utility under non-steady state heart rate conditions [35,53,54]. To date none of these latter approaches, although appealing, have been universally supported and accepted by most stakeholders.

## 7. QTc Syndromes

**Figure 6:**
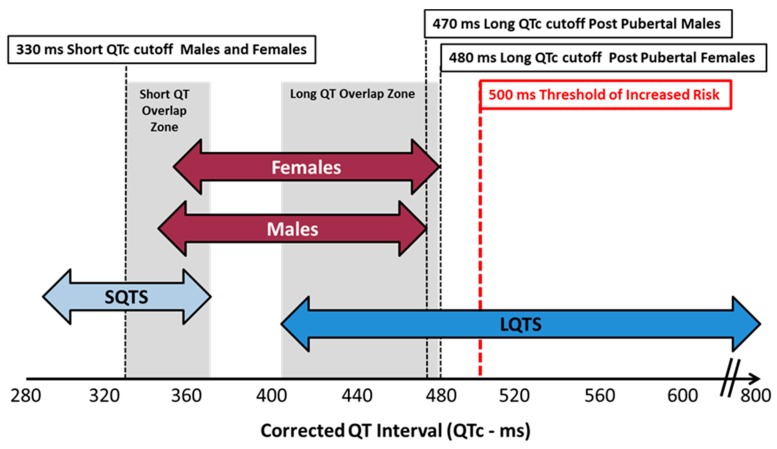
QTc values for congenital syndromes vs. normal healthy adults.

The screening of subjects for enrollment in early phase clinical studies is predicated on their satisfying certain QTc electrocardiographic inclusion and exclusion criteria universally aimed towards those with a prolonged rather than a shortened QTc interval (see Figure 6). These abnormalities in the QTc may be either acquired or inherited and occasionally the two conditions may coexist. It is therefore incumbent upon principal investigators involved in subject screening to recognize individuals who manifest criteria for either the long QTc or short QTc syndrome, exclude them from study participation, and refer them for further diagnostic evaluation when indicated.

While conventional analysis of the QTc interval as previously described remains the standard for screening of individuals for both inherited and acquired QTc prolongation, a somewhat novel and intriguing approach to detect altered ventricular repolarization is that of root mean squared electrocardiography (RMS ECG) [55]. The basis for development of this technology is that due to a low signal to noise ratio, the end of the T wave, being a low amplitude signal, may be difficult to detect especially with confounding U waves or in the presence of a short T-P interval. RMS-ECG is designed to address these concerns by measuring the QRS to T wave peak as a marker of mean ventricular APD and the RMS T wave width as a reflection of temporal dispersion of repolarization. The cellular basis of these measures has been confirmed in animal electrophysiologic studies but there is limited data in humans. [56] To address this shortcoming, Lux and coworkers derived RMS ECGs signals from 24 h Holter tracings in both subjects with moxifloxacin induced QT prolongation and in a pediatric population with congenital long QTc-2 syndrome. They demonstrated there was high precision in identifying individuals with delayed ventricular repolarization with less variability in QT values than that obtained from a standard lead II ECG. They concluded that this technique is best suited for newborns when rapid heart rates make distinguishing the end of the T wave from an encroaching P wave challenging. However, it has not been embraced or validated as a superior metric to conventional analysis in the healthy adult volunteer population and must await further studies in this group.

## 8. Long QTc Syndromes (LQTS)

### 8.1. Acquired LQTS

There are multiple medications which are known to prolong the QT interval and predispose to ventricular dysrhythmias. While cardiovascular antiarrhythmic drugs have the greatest notoriety, multiple classes of non-cardiac drugs such as antipsychotics, antibiotics, antidepressants, and antihistamines have been implicated and some of these have been withdrawn from the market due to their torsadogenic potential (Table 2). It is beyond the scope of this discussion to detail the more than 200 approved drugs which have the potential to affect the QT interval and predispose to TdP although this risk is comprehensively addressed and continually updated in the public access website CredibleMeds, www.crediblemeds.org.

QTc determination and I_Kr_ block are sensitive surrogate markers for arrhythmia risk but are not specific and have only a modest positive predictive value. There is no QTc value that definitively predicts the occurrence of TdP and the presence of a prolonged QTc interval does not in and of itself indicate that a malignant ventricular arrhythmia will occur. As a rough rule, it is estimated that the incidence of ventricular arrhythmias increases by 5%–7% for each 10 ms increment in the QTc value. Moreover, a QTc value >500 ms increases the risk of TdP by a factor of 2–3 fold while an increase of >60 ms from the baseline measurement is also thought to confer increased risk of arrhythmias [37]. Shah computed the relative torsadogenic risk in a group of noncardiac drugs and found that the risk increased incrementally when the placebo corrected QTc change from baseline exceeded 10 ms and had the highest potential for TdP when the QTc increased beyond 26 ms [57]. These data are in keeping with the current FDA thresholds of regulatory concern.

Prolongation of the QTc value and the occurrence of TdP in the clinical setting may be influenced by pharmacogenetic properties of the drug and its metabolite(s), an individual’s pharmacogenetic sensitivity and susceptibility, the compound’s lipophilicity, its distribution between plasma and myocardial tissue, and negative use dependence (augmented effects with bradycardia). Moreover, the proclivity to ventricular arrhythmias may be amplified by numerous physiologic conditions and drug interactions. In this regard, Zeltser et al. have demonstrated that in 71% of cases of drug related TdP two or more identifiable risk factors were consistently present [58]. A list of risk factors is highlighted in Table 3. Most prevalent amongst these risk factors is female gender as approximately 70% of cases are seen in women [22]. Also, the presence of polypharmacy and drug-drug interactions are frequently implicated in augmenting TdP risk with the cytochrome P450 system enzyme inhibitors most often being culprit. It is further recognized that individuals who develop TdP may have an underlying congenital LQT gene polymorphism or a genetic condition which alters drug metabolism either of which may predispose to this arrhythmia. Furthermore, in special populations such as cancer patients there is increased risk of drug interactions resulting in QTc prolongation. For example, greater prevalence is associated with breast and gastrointestinal cancer diagnoses as well as solid-based malignancy [59]. Finally, TdP may be influenced by an individual’s repolarization reserve [60], which is genetically determined and involves compensatory mechanisms that modulate unfavorable changes in depolarizing or repolarizing currents which may contribute to arrhythmogenesis.

### 8.2. Congenital LQTS 

Congenital prolongation of the QT interval comprises a group of inherited genetic mutations resulting in ion channelopathy that can lead to syncope, seizures and sudden death. To date there have been at least 13 different phenotypes related to 16 gene polymorphisms with some overlapping of clinical features [61,62,63]. Approximately 75% of cases are comprised of genetic mutations in *KCNQ1*, *KCNH2* and *SCN5A* which are termed long QTc syndrome (LQTS) 1, LQTS 2 and LQTS 3 respectively. The prevalence of congenital long QT syndrome is approximately 1 in 2000 individuals and is determined by measurement of resting QTc values as well as inspection of T wave morphology. The AHA proposed ULN QTcF values of >450 ms for adult males and >460 ms for adult females, it has been determined that the positive predictive value (PPV) for LQTS using these cutoffs is less than 1% and therefore they are not very helpful. Superior predictive recommendations for diagnosing LQTS have been promulgated by Ackerman and colleagues from their experience with the congenital LQTS registry and involve a point scoring set of diagnostic criteria [61], see Table 4. Key elements are that prepubertal individuals with a QTc value of >460 ms should be considered abnormal while for adult males a value of >470 ms and for females a value >480 ms increases the PPV of identifying this genetic disorder and may warrant referral for further diagnostic and possible genetic testing. Genetic testing would also be advised for any prepubertal individual with a QTc >480 ms on serial ECGs in the absence of any confounding factors. Testing is also appropriate for associated family members when genetic screening confirms the diagnosis of congenital LQTS in the affected individual. Finally, any suspected subject with a resting QTc value >500 ms dramatically increases the PPV for LQTS as well as dysrhythmias and mandates electrophysiology referral.

When evaluating subjects for LQTS a number of provisos need to be emphasized. Gene carriers may exhibit incomplete penetrance which may not be readily evident on a standard ECG while the presence of a long QTc by itself does not establish the diagnosis. Drugs, comorbid conditions and other secondary factors may be responsible for QTc prolongation and should be diligently sought. Measurement of the QTc should be undertaken when heart rates are between 50–100 bpm as bradycardia underestimates and tachycardia overestimates the QTc value which may lead to misdiagnosis. Repeat 12 lead ECG measurements may be necessary and computer-generated values should be confirmed by manual interrogation of either lead II or V5 while avoiding evaluation in the right precordial leads (V1-V2). T wave morphology should be carefully examined and the diagnosis of LQTS 2 suspected when bifid T waves are present with the second peak being taller than the first. Finally, a QTc value below the aforementioned AHA ULN thresholds, especially in the setting of a positive family history or supporting symptoms, does not exclude the diagnosis of LQTS (concealed LQT 1) and warrants further evaluation [64].

## 9. Short QTc Syndrome (SQTS)

### 9.1. Acquired SQTS

Similar to the long QTc syndrome, short QTc syndrome (SQTS) can either be acquired or congenital. In the 1990s it was postulated that short QT intervals in humans and some species of kangaroos may increase the risk of sudden cardiac death (SCD) [65,66]. Prior to 2000 when the short QT syndrome was first described, it was thought that shortening of the QTc interval was only seen secondary to a variety of metabolic, pharmacologic and pathologic conditions. These included hyperkalemia, hypercalcemia, acidosis and carnitine deficiency. Pharmacologic agents linked to QT shortening included digitalis, acetylcholine, ATP activators and catecholamines. Pathologic conditions that have been reported involve hypothermia, certain endocrine disorders, myocardial ischemia and post ictal states following epileptic seizures [62,65,66]. However, it is uncertain whether any of these predisposing factors routinely shortens the QT sufficiently to provoke any major clinical sequelae including syncope, seizures, malignant arrhythmias or SCD.

### 9.2. Congenital SQTS

In 1999, Gussak and coworkers first reported clinical cases of patients with shortened QTc intervals [65]; and in 2000 Gussak and Brugada [65], described a distinct inherited channelopathy in subjects who had a propensity for atrial and ventricular arrhythmias, syncope and SCD termed SQTS. SQTS may be sporadic or autosomal dominant in inheritance and is extremely rare with approximately 100 cases reported worldwide of the with a well-documented clinical syndrome. The mean age of presentation is around 30 years old and the majority of affected individuals manifest some symptomology the most common being palpitations, light headedness or syncope, and atrial fibrillation. Unfortunately, 28–40% of index cases may present with cardiac arrest. The prevalence of asymptomatic short QT values was reported in an 8-year study of 18,825 healthy young individuals from Seattle [67]. Twenty-six patients (0.1%) had a QTc <320 ms and 44 (0.2%) had a QTc <330 ms with a higher prevalence in males, athletes and those of African-Caribbean origin.

There are at least 6 gene defects that have been confirmed with this syndrome as illustrated in Table 5. The electophysiologic basis for this disorder appears to be missense mutations or genetic polymorphisms in genes that encode either potassium or calcium channels [65,66,68,69]. As a consequence, ventricular repolarization may be shortened by either a gain in function of the outward potassium channel or loss of function of the inward calcium channel. In either case, APD is reduced as is the effective refractory period of cardiac tissue resulting in a proclivity to both atrial and ventricular arrhythmias. Moreover, in response to the abbreviated repolarization time as a result of impaired calcium loading, echocardiographic findings of abnormally low global longitudinal strain and systolic dysfunction have recently been reported. The three predominant forms of SQT involve gain of function mutations of the potassium channel [66], as detailed in Table 5.

### 9.3. Criteria for Diagnosis SQTS

Due to the paucity of specialized referral sites that are capable of identifying nucleotide polymorphisms and the small number of cases in current registries, the criteria for diagnosis are somewhat varied. As such, there is some controversy as to what constitutes a short QTc value and screening based solely upon QTc results has been unrewarding. Population based studies and criteria from the Heart Rhythm Society consensus statement would suggest that a QTc interval ≤330 ms independent of gender or correction formulae is diagnostic even in the absence of any clinical history of cardiovascular events or confounding factors. Others have proposed that for females, a QTc ≤340 ms is also thought to be diagnostic without any history of prodromal symptoms. Lastly, a QTc value <360 ms in the presence of a documented missense mutation, a family history of SQT, a family history of sudden death in persons younger than 40 years of age or a history of atrial fibrillation, syncope or cardiac arrest are also considered diagnostic.

These guidelines are in keeping with those of Gollob et al. who have developed a point scoring scheme for SQTS predicated upon measurement of the QTc interval in conjunction with patient symptoms and a detailed family history [70]. As illustrated in Table 6, a score of 4 or greater confers a high probability for this diagnosis. To help establish the diagnosis, ECGs should be repeated on multiple occasions and typically when the heart rate is in the normal range ideally below 80 bpm, as correction formulae may underestimate the prevalence of this abnormality at higher heart rates leading to false negative diagnoses. Careful inspection of the ECG and associated rhythm strip may detect findings such as prominent U waves and peaked T waves, an absent ST segment, a prolonged Tpeak-Tend interval, or the inability to lengthen the QT interval as the heart rate slows. Similar to individuals with the long QTc syndrome, confounding factors should be excluded before conferring the diagnosis.

The presence of a shortened QTc interval as an isolated abnormality does not augur a poor prognosis as affected individuals have lived well into adulthood without any noteworthy clinical sequelae. As such, the role of genetic screening becomes unclear as documenting a missense mutation may not predict an adverse outcome. Nonetheless, in those persons with suspected SQTS, referral for additional diagnostic and possible genetic testing would appear warranted including a battery of tests to exclude latent structural heart disease. In those, however, with a well-documented symptomatic SQTS, genetic and electrophysiologic evaluation is required as is screening of first-degree relatives for risk stratification.

## 10. The Evolution of Regulatory Guidance Regarding Ventricular Repolarization 

ICH S7B [12] refers to the ***pre-clinical*** “Evaluation of the Potential for Delayed Ventricular Repolarization (QT Interval Prolongation) by Human Pharmaceuticals.” The elements of cardiac safety testing detailed in this publication were formulated based upon the knowledge that all drugs that were withdrawn from the market due to QT prolongation and TdP were associated with prolongation of phase 3 of the cardiac action potential primarily due to hERG channel block. As such, the major foci of the ICH S7B strategy included an in vitro assessment of ionic currents targeting a compound’s effect on I_Kr_ along with an in vivo QT assay in conscious or anesthetized non-human primates. Additionally, evaluation of action potential pharmacodynamics and proarrhythmic effects was suggested in isolated cardiac preparations the results of which were to be integrated with the above assays into a composite risk assessment for QT prolongation. All studies were to be conducted using Good Laboratory Practice (GLP) standards to comply with regulatory submission.

Of interest is the ability of preclinical assays to predict clinical QT effects as recently reviewed by Park et al. [71]. They examined hERG assays, APD and in vivo QTc effects obtained for 150 drugs in which ventricular repolarization had been evaluated in thorough QT trials [71]. They found that these assays showed a high false positive rate (poor sensitivity) but a low false negative rate (high specificity) at low concentrations with QTc as an endpoint. hERG and in vivo QTc data had better positive predictive values than the APD and were of moderate utility although they cautioned that these results can be variable dependent on drug concentration and testing conditions. Somewhat similar findings were reported by Kleiman and colleagues who computed the sensitivity and specificity of preclinical in vitro and in vivo studies to predict clinical QT prolongation [72]. Reduced sensitivity (30%–68%) across all assays was again noted while APD demonstrated a slightly higher specificity of 93% compared to the other studies at 85%.

ICH E14 [12] was designed to assess the ***clinical*** impact of pharmaceutical agents on the QT/QTc interval. This document recommended that most new chemical entities (NCE) that have systemic bioavailability be subjected to a randomized dedicated ECG trial involving normal volunteers termed a thorough QT (TQT) study. The purpose of this study is to determine if the novel agent under investigation demonstrates a threshold effect on the QTc interval. TQT study designs can be crossover, parallel or adaptive in configuration and recruitment of both male and female subjects, when appropriate, is encouraged. The conventional TQT study is typically comprised of 4 components: a therapeutic dose of the investigational compound, a supratherapeutic dose of the test compound, a placebo cohort and a positive control arm with a known QT prolonging agent, most often the antibiotic moxifloxacin, designed to confirm assay sensitivity. The parameters to be analyzed in these protocols include either a change from baseline of the heart rate corrected QT interval (ΔQTc) or the maximum time matched placebo adjusted change in heart rate corrected QT (ΔΔQTc) at any time point during the study. The threshold level of regulatory concern was set at a mean ΔΔQT effect of approximately 5 ms. This translates to a 10 ms upper bound of the one sided 95% confidence interval (CI) around the largest time matched mean effect of the baseline adjusted difference between placebo and study drug. This boundary is independent of whether it is breached by either the therapeutic or supratherapeutic dose of the compound. ΔΔQTc mean effects less than 10 ms provides reasonable assurance that the compound under investigation does not adversely alter ventricular repolarization. In this case, routine collection of on drug ECGs in later stage trials would be sufficient to monitor cardiac liability. In contrast, with pharmaceuticals that display a 10 ms (20 ms for oncology agents) or greater ΔΔQTc signal, expanded ECG surveillance in later stage development would almost always be required to characterize the QT effect of the agent. However, a positive signal should not automatically be interpreted as proarrhythmic nor should this constitute the sole grounds for the compound to be halted in development. In analyzing the pharmacodynamics effect of the test compound on the ECG, an important consideration is that while a mean change in a cohort(s) may not reach the 10 ms threshold, there may well be individuals within the study who have abnormally high changes in QTc exceeding 10 ms from baseline values. These “concealed outliers” would likely impact drug development including go/no-go decisions as well as the intensity of ECG surveillance in later stage protocols.

The TQT study is typically carried out late in stage II or early in stage III of drug development after pharmacokinetic and proof of concept information is available and is not limited to only NCE. It might also be undertaken for approved drugs when a different patient population is targeted, or a new dosing regimen or route of administration is being considered. In contrast, regulators have suggested that TQT studies are not routinely required for drug combinations when the individual components have previously been evaluated and did not manifest any QT liability. They also proposed that TQT trials are usually not indicated for large molecular weight biologic proteins or monoclonal antibodies which do not enter the cell and have minimal direct ion channel effects or agents which do not manifest any systemic bioavailability. Finally, during execution of these TQT studies, compliance with Good Clinical Practice (GCP) is necessary to ensure that data meets the regulatory standards for submission [43].

An additional topic that was recently addressed in a 2016 FDA document is the role of race and ethnicity in clinical trials [73,74]. Historically, TQT studies have focused on the QT liability or intrinsic risk of a compound independent of its effects on people of various races or ethnic persuasions and many trials are underrepresented by these subgroups. ICH E14 stated that ethnicity would likely not affect the results of a TQT trial. Although there is a scarcity of large-scale scientific studies, it is now recognized that different populations of individuals may have a genetic predisposition to being more ***susceptible and sensitive*** to a pharmaceutical’s effects either by attaining higher exposures or altering the drug’s disposition. This in turn may be due to heterogeneity in various alleles that code for different ionic channels or that impact metabolizing enzymes. To this point, one well known example of differential racial sensitivity is that of the agent BiDil [75], a combination of a nitrate and hydralazine. Despite considerable controversy, it was approved by the FDA in 2005 as there was a statistically significant improvement in all-cause mortality and first hospitalizations in self-identified Black subjects versus the overall study population. In contrast to TQT trials, early phase studies typically encompass small cohorts of subjects and therefore are underpowered to provide clinically meaningful results regarding ethnicity, race, and pharmacogenetic susceptibility and sensitivity. Therefore, efforts should be expended to recruit subjects of varying backgrounds especially as drug development proceeds into larger scale later phase studies. To this end, the FDA recommends that all Investigational New Drug (IND), NDAs and Biologic License Applications (BLAs) report tabulated demographic data per their 2016 guidance document [74].

## 11. Current FDA Guidance for Assessing QT Liability

TQT studies are resource intensive and expensive to conduct averaging between 1–4 million dollars depending upon study design. As such, all stakeholders have been interested in developing a more cost effective and equally informative scheme to assess a compound’s effect on the QT interval and its propensity to induce arrhythmias. To this end, there has been a paradigm shift towards a more comprehensive mechanistic approach to defining a compound’s cardiac risk and potential to produce TdP rather than only profiling its effects on the QT interval. This strategy entails a more robust preclinical and clinical evaluation of new compounds integrated with concentration QT (cQT) analysis in early phase clinical trials.

### 11.1. Comprehensive In Vitro Proarrhythmia Assay (CiPA) 

In 2013 an initiative was undertaken by industry, academicians and government regulators to develop a suite of preclinical and early clinical assays which would complement the ICH S7B guidance and provide an integrated assessment of arrhythmia risk. This initiative was known as the Comprehensive in vitro Proarrhythmia Assay (CiPA) [76]. In its most current iteration, it consists of the following 4 elements:

#### 11.1.1. Preclinical

Evaluation of ***multiple cardiac ion channels*** as a tool to profile risk when clinical studies demonstrate a modest QTc signal (typically <20 ms) and there is evidence of potassium channel/hERG block. In addition to a hERG kinetic score, the most important channels to be evaluated include L-type calcium (L-typeCa^2+^), late sodium (late Na^+^), slow potassium (I_Ks_), and occasionally other inward and outward voltage gated cations which may affect ventricular repolarization

Robust ***in silico computer modeling*** of a compound’s likelihood to cause TdP based upon data derived from ion channel studies. Compounds are to be classified as low, moderate, and high risk relative to a training and validation set of 28 approved reference drugs with known torsadogenic risk. A TdP metric score known as qNET has been devised to quantify risk into three subsets at exposures that are a multiple of the maximal concentration (Cmax). This model targets the assessment of early after depolarizations which are thought to be involved in the pathogenesis of TdP. It is not, however, designed to look at a compound’s direct effects on the cardiac action potential.

***Human induced pleuripotent stem cell cardiomyocyte*** preparations have been formulated to ascertain whether there are any unanticipated effects of a compound on ventricular cardiac tissue not documented by other investigations. This assay may be useful to uncover a NCE’s effects not identified by the in silico model, in situations where there is inadequate human exposure in early phase studies, or if there is discordant data from other preclinical assays.

#### 11.1.2. Clinical

**Figure 7:**
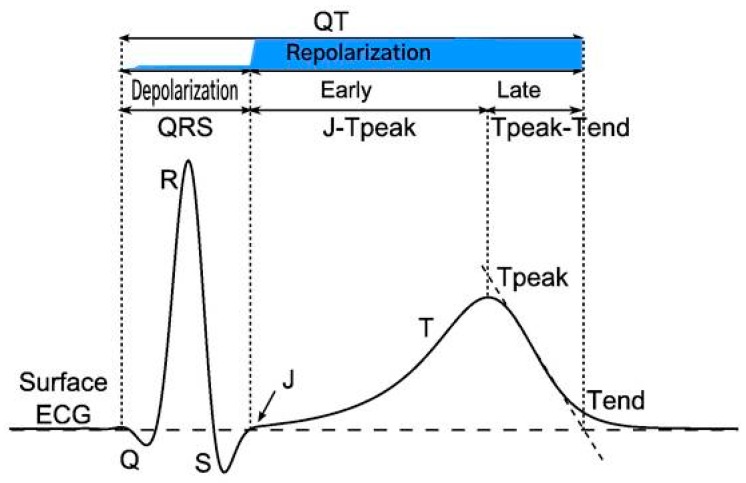
Principal waveforms and intervals of ventricular depolarization and repolarization on the surface ECG. Repolarization phase shaded in blue. Reproduced with minor edits from [77].

***Novel ECG biomarkers*** particularly evaluation of the early phase of cardiac repolarization, known as the J-T_peak_ interval, a mechanistic biomarker of multichannel ion block, which may mitigate cardiac risk when a modest (<20 ms) QT signal is observed in first in human studies. The J-T interval primarily corresponds to ventricular repolarization and is governed by the interplay of multiple voltage gated cations. This interval, in turn can be subdivided into the J-T_peak_ and the Tpeak-Tend segments (see Figure 7).

While I_Kr_ is the predominant outward ion channel influencing the length of ventricular repolarization and hence the overall J-T interval, inward currents mediated by calcium and late Na^+^ channel block are the principal factors responsible for the J-T_peak_c subinterval. It has been demonstrated that in compounds that prolong the QT interval due to single channel I_Kr_ block, an associated change of <9 ms in the J-T_peak_c value of the test compound is an indicator of late sodium multichannel ion block. [78]. This late sodium channel block has been postulated to attenuate the effects of I_Kr_ blockade and may therefore reduce the likelihood of ventricular arrhythmias.

The presence of multichannel ion block was first evaluated by Johanssen et al. [79], who published a paper in which they measured the QT, J-T_peak_c and Tpeak-Tend intervals in 4 drugs which were known to prolong the QT interval; ranolazine, quinidine, dofetilide and verapamil. They found that the pure hERG blocker dofetilide prolonged both the early and late components of the J-Tc interval and therefore had a higher risk of proarrhythmia. In contrast, the other three agents had a low risk of proarrhythmia and TdP due to multichannel ion block as evidenced by no appreciable prolongation of the corrected J-T_peak_c interval. In a follow up investigation to amplify their findings, these authors studied the ability to counteract the QT prolonging effects of the pure hERG blocker dofetilide by administering mexiletine and lidocaine which are two drugs known to affect late Na^+^ current [80]. They showed that these late Na^+^ channel blocking agents shortened the QTc by 20 ms and that this shortening was exclusively due to shortening of the J-T_peak_c interval.

Pursuant to these observations, at a recent presentation by Vicente [81], updated commentary was provided about the utility of measuring the early phase (J-T_peak_c) and late phase (J-T_peak_c-Tend) of the J-T interval as biomarkers that may modulate proarrhythmic concerns in the setting of a moderately positive QT signal and reduce the need for intensive ECG monitoring in late stage trials. To this end, open source code was released by the FDA using a vector magnitude function and software vendors including AMPS [82], Mortara and Philips have developed technology which offers measurement of these intervals based upon different mathematical models [83]. Although a meaningful disparity between the different vendors’ algorithms has not been evidenced, a number of critical elements in this approach have yet to be standardized which may affect the accuracy and utility of this biomarker. These include which computer algorithm to use; how best to determine the J point origin and the peak of the T wave especially when the T wave apex is not monophasic; in what lead(s) the J-T component intervals should be measured; and what is the optimal heart rate correction factor to utilize. It is unclear at this time if these metrics will be routinely required by regulators to assess QT liability or only be recommended to qualify risk whenever a modest QT signal is identified. Moreover, the decision as to which algorithm ECG core laboratory should use and validate is problematic given that the FDA’s posture is not to favor or endorse any specific methodology.

To date the FDA has recommended CiPA assays to 5 sponsors as part of their IND submissions although it is not a mandatory requirement that they be conducted or incorporated into drug protocols [84]. It therefore remains to be determined when CiPA information will be requested by government regulators, which elements of CiPA will be required, and who should be entrusted to provide these analyses. Furthermore, whether these assays should be pre-emptively undertaken by sponsors prior to IND submission and what would be the economic impact of adding these assays to drug development are both open questions for discussion. Hence, as additional CiPA assays are performed and the FDA databases this information, their precise role in a compound’s development scheme will be better delineated.

In summary, the major purpose of the CiPA initiative appears to be to improve product labeling regarding proarrhythmic potential, enable further development of drugs that previously may have been prematurely terminated due to perceived cardiac risk, and influence the intensity of ECG monitoring in late phase trials [85]. It may also prove helpful when uninterpretable TQT data due to heart rate changes are present, when the test compound cannot be given to healthy volunteers such as in oncology patients, and when inadequate or low exposure margins are seen in phase I trials. Its implementation may further be beneficial for pharmaceuticals that manifest significant heart rate changes and when there is confounding QT data from preclinical and early clinical studies. Finally, it should be viewed as a complementary approach to the ICH S7B guidance as hERG assays and animal telemetry observations are still required to assess cardiovascular safety.

### 11.2. Concentration QT Modelling

The second major development in QT liability assessment involves the use of exposure response analysis (ER) or concentration QT modelling (cQT) [86,87]. This type of analysis was under investigation in 2003 and became an integral part of TQT trials in 2006 albeit as a secondary analysis tool relative to the primary central tendency by time point approach known as the Intersection Union Test (IUT) as detailed in ICH E14. In 2008 the FDA drafted a guidance document elucidating the role of cQT in drug development [88] and in 2015 it became a key element in the FDA’s Q&A (R3) publication [12] that established it as a primary analysis tool to be applied predominantly in dose escalation studies as an alternative or “substitute” to conducting a large scale TQT study.

The emergence of cQT as a primary analysis modality is supported by a number of factors. Chief amongst these are the results of the Innovation and Quality Cardiac Safety Research Consortium (IQ-CSRC) SAD-like study [89], supporting the value of this approach in a small cohort of subjects, multiple computer simulation studies undertaken by stakeholders during the past 10 years, and a large body of knowledge garnered by conducting this analysis in hundreds of TQT trials. cQT has the distinct benefits of reducing resources and development timelines while enabling earlier go/no-go decisions which may obviate the need to undertake a large scale conventional TQT study. Additional advantages include facilitating extrapolation of a compound’s cardiac risk at exposures that may not have been evaluated but are clinically important, obtaining information in challenging populations such as oncology patients, and clarifying ambiguous results that may have been generated when the IUT was undertaken. The correlation between the findings of cQT analysis and those of the IUT applied to traditional QT studies is high when the highest clinically relevant exposure is achieved, and regulators have reviewed and accepted cQT with multiple IND submissions. cQT modeling has now become an important part of the discussion with sponsors as they plan their drug development programs and study designs.

When applying cQT analysis, most commonly, a pre-specified linear mixed effects model is used to compute the mean and two-sided 90% CI of ΔΔQTc at clinically relevant exposures covering concentrations that are therapeutic as well as those that might be seen with hepatic or renal impairment or in the presence of CYP enzyme polymorphisms. When the upper bound of the two-sided 90% CI for the QTc effect of a drug treatment as determined by exposure-response analysis is <10 ms, one can infer that the compound under investigation is not high risk and further testing beyond routine ECG surveillance in later stage development is usually not required assuming that the highest clinically relevant exposure has been attained. In contrast, when the cQT regression line manifests a positive slope that crosses the 10 ms threshold of regulatory concern, the compound is deemed to be at higher risk for proarrhythmia and may justify additional preclinical testing per the CiPA paradigm or more extensive ECG monitoring in later phase studies. Finally, when employing the cQT model, it is essential to avoid extrapolating data at concentrations that were not clinically observed and to ensure that tests for hysteresis are applied.

cQT is typically performed in SAD studies as the highest drug concentrations are usually achieved in these protocols and the probability of reaching a targeted exposure level is greater than that which would be seen in multiple ascending dose (MAD) protocols. Incumbent in the SAD trials is that there are a sufficient number of subjects to provide adequate power to exclude a false negative result. This typically includes 3 or more cohorts of 6–9 subjects on active drug and at least 2 subjects per cohort receiving placebo. However, there are situations when cQT in SAD investigations may be challenging and the preferred strategy in these cases would be to incorporate cQT analysis in a MAD design. These scenarios include drugs which may be time released such that the model fails to capture a sufficient range of drug exposures, drugs which exhibit significant hysteresis and heart rate variability, and for drugs in which hERG blocking effects of the parent moiety and an associated active metabolite may not be fully manifest in a single dose escalation protocol. Moreover, in circumstances when the investigational agent has non-linear kinetics, or when there may be a long half-life, major metabolites or significant drug accumulation, the SAD study may not have enough subjects for meaningful analysis. In these cases, data from the SAD investigation may be combined with pharmacokinetic (PK) and pharmacodynamic data from a complementary MAD study assuming that clinical conduct of both trials is homogeneous and ECGs are rigorously collected, of high quality, and reviewed in a consistent and blinded manner so as to minimize data variability [43].

The decision to undertake cQT in early phase trials may be influenced by budgetary constraints although the expense associated with this strategy is significantly less than that of a dedicated TQT trial. In these situations, several cost effective alternatives may be attractive. One would be to only analyze the PK/QTc relationship in the highest dose cohorts while another approach would be to collect Holter recordings for each cohort, and store and save the information for future analysis, as most NCE will not proceed beyond phase II in development.

An important issue of regulatory concern is whether or not a positive control to document assay sensitivity is required for exposure response analysis to reduce the likelihood of false negative results. The original ICH E14 document stated that “the confidence in the ability of the study to detect QT/QTc prolongation can be greatly enhanced by the use of a concurrent positive control group (pharmacological or non-pharmacological) to establish assay sensitivity.” Current guidance for cQT does not mandate the use of a pharmacologic positive control assuming the drug achieves an adequate exposure margin defined as twice the highest clinically relevant exposure which is generally higher than the supratherapeutic dose observed in conventional TQT trials. This exposure represents a worst-case clinical scenario accounting for both intrinsic and extrinsic factors that can affect drug metabolism. These include renal or hepatic impairment, drug-drug interactions, gender, age, food and metabolic enzymes that impact drug disposition. As such, the margin for patient safety and the likelihood of false negative responses are optimally addressed by analyzing data bracketing the Cmax, the highest clinical exposure, and at the timepoint when a multiple of the highest relevant clinical exposure occurs. In the event that a 2X exposure margin is not reached, then a positive control with moxifloxacin administered to 20–24 subjects has been proposed to establish assay sensitivity.

Alternatively, a non-pharmacologic approach to assess assay sensitivity known as “bias metric” [90], is currently undergoing examination and is most suitable in cases where the exposure margin falls just short of waiving the need for a positive control. This approach uses the slope of Bland Altman plots to address any potential bias between fully automated computer-generated QT measurements and those that are procured from the core laboratory algorithm used in the primary analysis. Darpo et al. published their results using this metric for 5 drugs with known QT prolonging effects that were also administered in the IQ-CSRC study [91]. When bias severity was greater than −20 ms over a range of QTc values of 100 ms or greater, then the ability to predict the QT effects of the five studied drugs failed. Contrariwise, when there was a QTc difference of <−10 ms bias severity, then the chance of a false negative finding was lowered to less than 5%. They concluded that a metric of bias severity “has to be included” in all reported QTc information obtained in early phase studies using exposure response analysis to minimize the risk of false negative results and ensure accuracy and consistency of fiducial measurements. To date, this recommendation has not been routinely fostered by government regulators who view its application on a case by case basis where it seems to have the greatest impact with drugs that demonstrate some degree of QT prolongation. Other non-pharmacologic approaches that have been proposed to establish assay sensitivity include the effects of standardized meals as advocated by Taubel [92], and the role of positional changes reported by Wheeler [93]. Neither of these have gained traction within industry and all non-pharmacologic approaches to determine assay sensitivity await further testing and validation before they become viable and accepted alternatives to moxifloxacin.

From December 2015–November 2016, the QT-Interdisciplinary Review Team (QT-IRT) reviewed 25 proposals for cQT analysis submitted under the aegis of ICH E14 (Q&A R3) [94], 18 of which were early phase SAD/MAD study designs including some where data was pooled from the separate trials. The reviewers accepted 11 proposals while rejecting 14 of the submissions with the predominant reason for rejection being inadequate exposure margins or an inadequate modeling analysis plan. In a more recent update of 16 study designs of non-oncology compounds, the QT IRT accepted 14 as a substitute for conducting a TQT study while rejecting 2 [84]. Those that were rejected were once again primarily due to inadequate exposure margins. Other possible reasons for protocol modification or outright rejection have included failure to sample at Cmax, failure to sample for a sufficient time out to 24 h, failure to consider the impact of metabolites and inadequate justification for pooling of data from multiple studies. Moreover, when submitting a proposal involving cQT, there are other important elements to consider in the study design that might prompt rejection if omitted. These entail exploratory plots to test model assumptions, utilizing a linear mixed effects model or other alternative models such as Emax, and comprehensive treatment−placebo difference calculations. While not all protocols are mandated to be submitted for review to the FDA’s cardio-renal division and QT-IRT, submission of all first in human drug proposals should be considered if there are concerns about study design, modelling or exposure levels. This review process typically encompasses 30–60 days following submission and should be incorporated by sponsors into their development timelines.

## 12. Commentary

The 10 ms threshold of regulatory concern, while having been successful in preventing any new drugs that are torsadogenic from coming to market since 2005, is not typically a value that would prompt intervention by clinicians, particularly if the calculated QTc after drug administration remains well within the normal range. Moreover, depending upon the specialty of practice, clinicians may not be aware that certain compounds have a risk of inducing rhythm disturbances and, more importantly, that drug combinations in the setting of underlying disease or electrolyte abnormalities may be synergistic in increasing the risk of an arrhythmic event. To add, even when there is documented and known QT liability for a drug, clinicians may not procure pre and post treatment ECGs to monitor the QTc interval. For example, a study by Choo et al. from the UK surveyed the prescribing practice of hospital based cardiologists and general practitioners over 6 months involving drugs which carried a risk of prolonging the QTc interval [95]. A baseline prolonged QTc was recognized by only 14% of cardiologists and 6% of general practitoners. Of the 4133 patients in the study, 22 had QTc values >500 at baseline but only 2 of these were identified prior to drug dosing. Surprisingly, despite manifesting a prolonged QTc at baseline, a significant number of subjects (37.8%) were nonetheless prescribed QTc prolonging drugs and only 8% had a follow up ECG at 48 h. Broszko and Stanciu surveyed a small group of psychiatrists from a university program and found that despite American Psychiatric Association (APA) recommendations [96], a large number of the physicians did not routinely screen for syncope, family history of sudden death, heart disease or congenital long QTc syndromes. Moreover, obtaining ECGs prior to initiating treatment was performed much less frequently in the outpatient cohorts compared to the inpatient population. This study may well be representative of other subspecialties underscoring the need for all practitoners to be cognizant of a drug’s potential for producing arrhythmias prior to initiating therapy coupled with careful cardiovascular screening. To aid in this endeavor, the website CredibleMeds is an important and user-friendly reference in the public domain as it maintains a comprehensive list of medications that may be linked to TdP and classifies them as “known risk, possible risk and conditional risk”. It also has a category of drugs to be avoided in the setting of congenital long QTc syndromes and the drug lists are continually updated as new information becomes available.

Based upon the aforementioned information, it is evident that prescribers including cardiac specialists fall short in appropriate surveillance for QT liability and need to be more diligent. This would entail performing a careful history for cardiovascular disease and obtaining a baseline ECG if indicated. While it is incumbent that regulatory agencies focus on public safety including the challenge of weighing a compound’s benefit versus risk during the approval process, more attention needs to be directed towards practitioners to increase their awareness about drugs with known QTc effects often embedded in drug insert black box warnings. To illustrate this need, in a survey of roughly 5 million prescriptions in the UK, 23% of patients were prescribed a drug that the prescriber did not recognize could prolong the QT interval [97]. Accordingly, the mission of protecting the public is a shared responsibility involving both government regulators and health care providers and additional safeguards should be instituted to ensure patient safety when drugs that may affect cardiac repolarization are administered.

As an aid in this endeavor, computer software programs that identify adverse drug responses and potential drug interactions based upon individual patient demographic and laboratory data need to be developed and universally adopted to optimally safeguard patients from QT liability. To this end, the Mayo Clinic and University of Indiana have independently implemented clinical data support systems (CDSS) in an effort to alert pharmacists and physicians to medications which may predispose to arrhythmias [98]. For example, at Indiana University Medical Center, 2400 electronic records from coronary care unit patients were interrogated and a QTc alert risk score was developed based upon a QTc >500 ms or a change from baseline of >60 ms. Using this approach, they demonstrated that the risk of exceeding either of these thresholds could be reduced for both cardiac and non-cardiac drugs that have been associated with QT prolongation and TdP. Unfortunately, however, in the majority of cases when alerts were generated by the CDSS systems, prescribers and pharmacists ignored the alerts thereby compromising the favorable impact of these systems on reducing the likelihood of serious ventricular arrhythmias.

Despite these shortcomings, the further development of comprehensive CDSS that are integrated with laboratory and clinical proarrhythmic risk factors should be pursued in combination with continuing education for those entrusted with dispensing and prescribing medications. The development of wearable smart devices or handheld software applications that can monitor a patient’s rhythm, assess the QT value, and transmit an alert would be useful for risk stratification and surveillance. Moreover, as genetic screening becomes more widespread and cost effective, this information could be integrated into these computer alert platforms thereby allowing persons with congenital QT syndromes to be identified sooner. This in turn would translate into heightened awareness of all parties involved and permit earlier prophylactic intervention so as to reduce patient morbidity and mortality. Future strategies to mitigate arrhythmia risk may include stereoselective and molecular engineering of new compounds to reduce undesirable arrhythmogenic effects, the development of agents that shorten the QT interval such as hERG activators that could be targeted to counteract the QT prolonging activity of drugs, and compounds that can shorten APD via blockade of the Ca^2+^/Na^+^ exchanger. However, as cautioned by Malik [99], the science to support these approaches is yet to be substantiated and there is the possibility that QT shortening compounds may paradoxically create unwanted heterogeneity of repolarization and arrhythmogenicity in some cases.

## 13. Conclusions

It is evident from the foregoing discussion that the QTc interval, despite its modest positive predictive value for TdP and ventricular arrhythmias, continues to occupy a central focus of regulators as a surrogate marker for proarrhythmic risk during drug development. However, there has been an evolution in how stakeholders are approaching cardiac liability as embodied by the CiPA paradigm with its advanced preclinical assays, and the emergence of cQT as a primary analysis tool in dose escalation studies. Taken together, these paradigms offer a more mechanistic and cost-efficient approach to assess arrhythmia risk than that afforded by a conventional TQT trial.

While the QTcF correction formula remains the reporting standard for regulatory review, there is a lack of consensus as to what constitutes a normal value and it is recommended that protocol specific thresholds are chosen balancing the elements of subject safety against recruitment challenges. When QTcF does not adequately correct for heart rate effects, determination of an individual QTcI either by a full Day-1 acquisition of QT-RR pairs or following autonomic maneuvers such as postural change has been suggested. While the measurement of the QT interval can be problematic, the use of computer assisted algorithms to initially place fiducial markers followed by manual review and adjudication by experienced pharma QT expert readers would ensure that the precision and accuracy necessary to profile a compound’s effects on ventricular repolarization are realized. Equally important is the recognition of both acquired and congenital LQTS and SQTS polymorphisms so as to permit appropriate triage of these individuals for diagnostic evaluation and testing. It is also critical to exclude them from enrollment in pharmaceutical studies that may aggravate an underlying predisposition to alter ventricular repolarization leading to serious ventricular arrhythmias and TdP. Finally, continued development and acceptance of user-friendly interfaces for health care professionals which allow real time access to drug information and clinical data are promising tools to ameliorate the risk of QT associated dysrhythmias.

## Figures and Tables

**Table 1:** Popular Correction Formulae for QT values.

Formula Name	Equation	Reference
Bazett	QTcB = QT/RR^1/2^	[20]
Fridericia	QTcFri = QT/RR^1/3^	[21]
Framingham	QTcFra = QT + 0.154 (1 − RR)	[22]
Hodges	QTcH = QT + 0.00175 ([60/RR] − 60)	[23]
Rautaharju	QTcR = QT − 0.185 (RR − 1) + k (k = + 0.006 s for men and + 0 s for women)	[24]
Individual	QTc_i_ = QT_i_/RR_i_^bi^ multiple mathematical formulae have been proposed (see below)	
Dmitrienko	QTcDMT: mixed effects modeling formula	[25]
Population based	QTcP = QT/RR^b^ off treatment baseline ECGs	
Van de Water	QTc = QT − 0.087{(60/HR) − 1}	[26]
Other	Cross validated spline correction factor which is independent of HR	[27]

Table adapted from [28].

**Table 2:** List of drugs withdrawn from the market due to torsadogenic risk.

Drug	Therapeutic Class	Year Withdrawn from Market
Prenylamine	Angina	1988
Terodiline	Urinary Incontinence	1991
Sparfloxacin	Antibiotic	1996
Terfenadine	Antihistamine	1998
Sertindole	Antipsychotic	1998
Astemizole	Antihistamine	1999
Grepafloxacin	Antibiotic	1999
Cisapride	Prokinetic	2000
Droperidol	Antipsychotic	2001
Levomethadyl	Opiate Dependence	2003
Propoxyphene	Analgesic	2015

**Table 3:** Risk factors for Torsades de Pointes.

Risk Factor
QTc > 500 ms
Use of QT prolonging drug(s)
Abnormal repolarization morphology on ECG: notching of T waves, long Tpeak-Tend
Underlying heart disease: heart failure or myocardial infarction
Female gender
Hypokalemia
Hypomagnesemia
Hypocalcemia
Hypothyroidism
Advanced age
Bradycardia
Premature contractions producing short-long-short cycles
Impaired hepatic clearance of drugs
Diuretic use
Renal failure
Latent congenital LQTS polymorphisms
Abnormal repolarization reserve
Combinations of 2 or more risk factors

Adapted from [22,37].

**Table 4:** Diagnostic criteria for long QT syndrome (LQTS).

Category.	Criteria	Score
Electrocardiogram	QTcB interval:	
	≥480 ms	3
	460–479 ms	2
	450–459 (male) ms	1
	QTcB 4th minute of recovery from exercise stress test ≥480 ms	1
	Torsade de Pointes	2
	T-wave alternans	1
	Notched T-wave in three leads	1
	Low heart rate for age (below the 2nd percentile)	0.5
Clinical History	Syncope:	
	With stressful activity	2
	Without stressful activity	1
	Congenital deafness	0.5
Family History	Family members with definite LQTS	1
	Unexplained sudden cardiac death below age 30 among immediate family members	0.5
**Diagnosis**	**Probability of LQTS**	**Sum of Score**
	Low	≤1
	Intermediate	1.5 to 3
	High	≥3.5

Adapted from: Schwartz and Ackerman [61]. LQTS is diagnosed in the presence of an LQTS risk score ≥3.5 in the absence of a secondary cause for QT prolongation and/or in the presence of an unequivocally pathogenic mutation in one of the LQTS genes or in the presence of a corrected QT interval for heart rate using Bazett’s formula (QTc) ≥500 ms in repeated 12- lead electrocardiogram (ECG) and in the absence of a secondary cause for QT prolongation. In addition, LQTS can be diagnosed in the presence of a QTc between 480 and 499 ms in repeated 12-lead ECGs in a patient with unexplained syncope in the absence of a secondary cause for QT prolongation and in the absence of a pathogenic mutation.

**Table 5:** Genes Associated with short QT syndrome (SQTS).

SQTS Subtype.	Gene Name	Protein Name	Function	SQTS Mechanism
SQT-1	*KCNH2*	Kv11.1	α-subunit I_Kr_	Gain-of-function
SQT-2	*KCNQ1*	Kv7.1	α-subunit I_Ks_	Gain-of-function
SQT-3	*KCNJ1*	Kir2.1	α-subunit I_K1_	Gain-of-function
SQT-4	*CACNA1C*	Cav1.2	α-subunit I_L,Ca_	Loss-of-function
SQT-5	*CACNB2*	Cavβ2	β2-subunit I_L,Ca_	Loss-of-function
SQT-6	*CACNA2D1*	Cavδ1	δ1-subunit I_L,Ca_	Loss-of-function

Adapted from: [66].

**Table 6:** Diagnostic criteria for SQTS.

Category	Criteria	Score
Electrocardiogram	QTc interval:	
	<370 ms	1
	<350 ms	2
	<330 ms	3
	Jpoint-Tpeak interval <120 ms	1
Clinical History	History of sudden cardiac arrest	2
	Documented polymorphic VT or VF	2
	Unexplained syncope	1
	Atrial fibrillation	1
Family History	Family member with high-probability SQTS	2
	Family member with autopsy-negative sudden cardiac death	1
	Sudden infant death syndrome	1
Genotype	Genotype positive	2
	Mutation of undetermined significance in a culprit gene	1

Adapted from Gollob et al. [70]. A score of 4 or greater confers a high probability of confirming the diagnosis of short QT syndrome.

## Abbreviations

APD	action potential duration
CiPA	comprehensive in vitro proarrhythmia assay
Cmax	maximal concentration
cQT	concentration QT modelling
ECG	electrocardiogram
FDA	Food and Drug Administration
hERG	human ether a-go-go related gene
ICH	International Conference on Harmonization
LQTS	long QT syndrome
MAD	multiple ascending dose
NCE	new chemical entity
QTc	corrected QT
SAD	single ascending dose
SCD	sudden cardiac death
SQTS	short QT syndrome
TdP	Torsades de Pointes
TQT	thorough QT
